# Proximal Sessile Serrated Adenomas Are More Prevalent in Caucasians, and Gastroenterologists Are Better Than Nongastroenterologists at Their Detection

**DOI:** 10.1155/2017/6710931

**Published:** 2017-12-18

**Authors:** Malav P. Parikh, Sujit Muthukuru, Yash Jobanputra, Kushal Naha, Niyati M. Gupta, Vaibhav Wadhwa, Rocio Lopez, Prashanthi N. Thota, Madhusudhan R. Sanaka

**Affiliations:** ^1^Department of Gastroenterology and Hepatology, Cleveland Clinic Foundation, 9500 Euclid Avenue, Cleveland, OH 44195, USA; ^2^Department of Internal Medicine, Presence Saint Francis Hospital, 355 Ridge Ave, Evanston, IL 60202, USA; ^3^Department of Biostatistics and Quantitative Health Sciences, Cleveland Clinic Foundation, 9500 Euclid Avenue, Cleveland, OH 44195, USA

## Abstract

**Background and Aim:**

Proximal sessile serrated adenomas (PSSA) leading to colorectal cancer (CRC) represent an alternate pathway for CRC development. In this study, we aim to determine the prevalence of PSSAs and the impact of patient, colonoscopy, and endoscopist-related factors on PSSA detection.

**Methods:**

Patients ≥ 50 years of age undergoing a screening colonoscopy between 2012 and 2014 were included. Detection rates based on patient gender, race, colonoscopy timing, fellow participation, bowel preparation quality, and specialty of the endoscopist were calculated. *t*-tests were used to compare detection rates and a multivariate-adjusted analysis was performed.

**Results:**

140 PSSAs were detected from 4151 colonoscopies, with a prevalence of 3.4%. Detection rate was higher in Caucasians compared to African-Americans (AA) (3.7 ± 4.1 versus 0.96 ± 3.5; *p* < 0.001). Gastroenterologists detected more PSSAs compared to nongastroenterologists (3.9 ± 3.5 versus 2.2 ± 3.0; *p* = 0.028). These findings were still significant after adjusted multivariate analysis. The rest of the factors did not make significant difference in PSSA detection rate.

**Conclusions:**

PSSAs are more prevalent in Caucasians compared to AAs. Racial difference in prevalence of PSSAs is intriguing and warrants further investigation. Gastroenterologists have a significantly higher PSSADR compared to nongastroenterologists. Educational measures should be implemented in nongastroenterologists to improve their PSSA detection rates.

## 1. Introduction

In recent times, the serrated adenoma-neoplasia pathway has emerged as an alternative mechanism to the conventional adenoma-carcinoma pathway for the development of colorectal cancer (CRC) [1–6]. This alternate pathway can account for almost 15–20% of the incident CRCs and majority of the interval cancers after a screening colonoscopy [7, 8]. These tumors have a high frequency of BRAF mutations, microsatellite instability, and hypermethylation of genes [9–11]. Serrated lesions are often difficult to detect during a screening colonoscopy as they are flat or sessile, have an indiscriminate edge, may be covered by a mucous cap, and are located mostly in the proximal colon [12, 13].

Originally, all the serrated lesions were believed to be hyperplastic polyps (HP) with no malignant potential; however, now, it has been identified that few subtypes of serrated lesions do harbor malignant potential [3–6, 14]. According to the world health organization (WHO), serrated lesions are classified into (1) hyperplastic polyps; (2) sessile serrated adenomas (SSA) with or without dysplasia; and (3) traditional serrated adenomas (TSA) [15]. HPs are essentially benign. TSAs have malignant potential; however, they are uncommon. Hence, SSAs, which are located mostly in the proximal colon, appear to be the principal precursor lesions leading to CRC via the alternate pathway. It has been shown that serrated polyp detection rate is dependent on the endoscopist, experience of the pathologist, and colonoscopy withdrawal times [13, 16, 17]. However, there is only limited data on the impact of patient-related factors such as gender and race or endoscopy-related factors such as quality of bowel preparation, timing of the procedure, fellow participation, or specialty of the endoscopist, on the proximal sessile serrated adenoma detection rate (PSSADR) [18, 19]. The aim of this study was to determine the prevalence of proximal SSAs (PSSA) in an average risk screening population and the effect of various patient and endoscopy-related factors on PSSADR.

## 2. Materials and Methods

This is a retrospective chart review study performed at the Cleveland Clinic Foundation, Cleveland, OH, USA. Patients aged 50 years and older with average risk factors for CRC who underwent a complete screening colonoscopy between January 1, 2012 and December 31, 2014 were included in the study. Institutional review board at the Cleveland Clinic Foundation approved the study. Demographic details including patient age, gender, and race were collected. Endoscopy and pathology reports from all included colonoscopies were reviewed. A screening examination was defined, as a colonoscopy for which there was no surveillance or diagnostic indication. The proximal colon was defined as inclusive of the cecum, ascending colon, transverse colon, and splenic flexure. Distal to this was defined as distal colon. Adenoma detection rate (ADR) was defined as the proportion of screening colonoscopies in which at least one histologically confirmed colorectal adenoma was detected. Proximal serrated polyp detection rate (PSPDR) was defined as the proportion of colonoscopies in which at least one proximal serrated polyp (inclusive of HP, SSA, and TSA) was detected. PSSADR was defined as the proportion of screening colonoscopies in which at least one PSSA was detected. Overall (proximal + distal) sessile serrated adenoma detection rate (SSADR) was defined as the proportion of screening colonoscopies in which at least one SSA was detected. PSSADR according to patient gender and race, timing of colonoscopy, quality of bowel preparation, fellow participation, and endoscopist specialty were calculated. Overall ADR, SSADR, and PSPDR were also calculated.

Timing of the procedure was defined as morning (before 12 : 00 pm) or afternoon (after 12 : 00 pm) based on the procedure start time. Quality of bowel preparation was determined as per the Aronchick scale (excellent, good, adequate, inadequate, or poor). Patients with inadequate and poor bowel preparation were excluded from the analysis. Participation of a trainee fellow along with the attending physician during the procedure was noted. Colonoscopy was performed by gastroenterologists, general surgeons (GS), colorectal surgeons (CS), and one primary care physician (PCP). GS and CS were grouped together as nongastroenterologists. Colonoscopy performed by the PCP was not included in the study due to a very small number (*n* = 1). Individual endoscopists with less than 10 procedures each were also excluded. All the cases were reviewed by one of the 15 subspecialty gastroenterology pathologists. Educational interventions are regularly implemented to improve serrated polyp detection and standardize classification to minimize any variation in PSSADR due to pathology interpretation. All the screening colonoscopies were performed in an academic medical setting.

### 2.1. Statistical Analysis

Data are presented as mean ± standard deviation, median (25th, 75th percentiles) or frequency (percent). All endoscopist-level data was calculated from patient-level data. Paired *t*-tests were used to compare PSSADR by patient gender, race, presence of fellow, and timing of the procedure. Linear mixed models were used to compare detection rates among patients with excellent, good, and adequate preparation; a random effect was modeled to account for endoscopist. Also, detection rates were compared between physician specialty using *t*-tests.

A multinomial regression analysis was performed to check for the association between dependent and independent variables. All analyses were done using SAS (version 9.4, The SAS Institute, Cary, NC), and a *p* < 0.05 was considered statistically significant.

## 3. Results

A total of 4151 patients underwent screening colonoscopy over the study period. Average patient age was 60.0 ± 7.7 years, 53.2% (*n* = 2207) patients were females and Caucasians comprised 80.3% (*n* = 3334) of the entire cohort (Table 1). A total of 84 endoscopists performed the colonoscopies, with an average of 49.41 (22–65) procedures per endoscopist. 54 endoscopists (63.5%) were gastroenterologists, and 30 (36.5%) were nongastroenterologists. Fellows participated in 8.8% (*n* = 367) of the procedures. Majority of the colonoscopies were performed in the morning (70.3%). As per the Aronchick bowel preparation scale, majority of the colonoscopies were classified as having good quality bowel preparation (64.4%; *n* = 2675) (Table 2). A total of 140 PSSA were detected among the 4151 screening colonoscopies with a prevalence of 3.4% and a mean PSSADR of 0.04 ± 0.25 per patient. Overall and gender-specific ADR, PSPDR, PSSADR, and SSADR are shown in Table 3.

Overall PSSADR was significantly higher in Caucasians as compared to African-Americans (AA) (3.7 ± 4.1 versus 0.96 ± 3.5; *p* < 0.001). This was seen in both males (4.2 ± 6.3 versus 1.10 ± 5.0; *p* = 0.003) and females (3.4 ± 5.1 versus 0.88 ± 3.3; *p* < 0.001). Patient gender, timing of the procedure, quality of the bowel preparation, and fellow participation had no effect on PSSADR. Gastroenterologists were more likely to detect PSSA compared to nongastroenterologists (3.9 ± 3.5 versus 2.2 ± 3.0; *p* = 0.028) (Table 4). ADR was also significantly higher for gastroenterologist as compared to the surgeons (28.8 ± 10.5 versus 22.1 ± 10.8; *p* = 0.007) (Table 5). When assessing the detection rates for general surgeons versus colorectal surgeons, a trend towards higher ADR, PSPDR, PSSADR, and SSADR was noted for colorectal surgeons as compared to general surgeons; however, this was statistically not significant (Table 6). Data should be interpreted carefully as there were fewer general surgeons (*n* = 9) in the study as compared to colorectal surgeons (*n* = 21). A multinomial regression analysis was performed to check for the association between dependent and independent variables. Caucasian race and gastroenterologists performing the colonoscopy were associated with increased PSSADR even after adjusting for all the independent variables, and this was statically significant (Caucasians versus AAs: *p* = 0.003 and gastroenterologist versus nongastroenterologists: *p* = 0.001).

## 4. Discussion

CRC is the second leading cause of cancer-related deaths in the US and the third most common cancer in both men and women. A total of 51,651 deaths were reported due to CRC in 2014 in the US [20]. Traditionally linked only to the adenoma-carcinoma sequence, it is now well known that CRC can also arise from an “alternate,” serrated neoplasia pathway, SSA being the chief precursor lesion [1–6]. We specifically studied a cohort of 4151 average risk patient population undergoing screening colonoscopy that is representative of the general US population, enabling us to identify important patient- and endoscopy-related factors that affect PSSADR.

In average risk screening patients, the reported SSA prevalence ranges from 2% to 7% [1, 21]. Other investigators have studied PSPDR, and it is reported to range from 1% to 22% [13, 16, 22]. In our study, the prevalence for PSSA was 3.4% and PSPDR was 6.2% (inclusive of HPs, SSAs, and TSAs). Patient gender was not associated with significant differences in the PSSADR. These findings are similar to prior studies [1].

### 4.1. Race and PSSADR

According to the 2015 US census bureau [23], racial composition of the US population was 77.1% Caucasians and 13.3% AAs. Our study population was similar and largely reflective of this racial distribution, where Caucasians were 80.3% and AAs were 15.2%. In our study, after adjusting for all the independent variables, PSSADR was significantly higher in Caucasians compared to AAs (*p* = 0.003). This was true for both Caucasian males and females. PSPDR (inclusive of HPs, SSAs, and TSAs) was also higher in Caucasians compared to AA, similar to prior studies [24]. Wallace et al., in a study of uninsured and low-income population showed similar results, where Caucasians were noted to have a higher prevalence of SSAs and any serrated polyps compared to AAs [25]. A study comparing the prevalence of SSAs in Caucasian and Chinese populations also found that SSAs were more common in Caucasians than Chinese (7% versus 2%; *p* = 0.001) [21]. This would imply that “serrated pathway” might be largely responsible for incident and interval CRC in Caucasians compared to other race or ethnic groups [21, 25, 26].

Adenomas developing within different carcinogenic pathways (e.g., conventional or serrated) may evolve into invasive carcinomas with differing prognostic features. MSI-H sporadic cancers evolve from the precursor lesions of the serrated pathway [15], which is observed to be more common in whites than blacks. In accordance with this, a population-based study comparing MSI-H cancer by race has shown whites to have a higher prevalence of MSI-H cancers as compared to blacks [27].

On the contrary, studies have reported a more proximal distribution of adenomas in AAs than whites [27–30]. Also, AAs are at a higher risk (OR 1.15; 1.03–1.29) for detection of large proximal polyps as compared to whites [31]. Further, a large study evaluated the relationship of race and the location of CRC and found that AAs were significantly more likely than Whites to develop proximal CRC [32]. Given the rarity of serrated polyps in AAs, serrated pathway does not seem to contribute significantly towards the occurrence of proximal CRC in AAs and it is possibly related to adenoma-carcinoma pathway.

### 4.2. Timing of Colonoscopy and PSSADR

A study by Sanaka et al. showed that adenoma detection (ADR) rates were significantly higher for colonoscopies performed in the morning as compared to in the afternoon [33]. Similarly, in a study by Chan et al, more polyps were detected in patients receiving colonoscopies early in the morning and adenoma detection rate reduced as the day progressed [33, 34]. Operator fatigue is proposed as a probable reason for reduced colonoscopy efficiency in the afternoon. On the contrary, a study, which included more than 100,000 screening colonoscopies in fact, found that afternoon procedures were 1.14 times more likely to detect advanced lesions as compared to the morning colonoscopies [35]. There is paucity of studies that assessed the impact of colonoscopy timing on detection of serrated lesions. In our study, PSSADR did not differ significantly between morning and afternoon procedures [36]. Hetzel et al. noted similar results in a study, where SSA detection was not associated with the hour of the endoscopy [1].

### 4.3. Quality of Bowel Preparation and PSSADR

Detection of conventional adenomas seems clearly linked to better quality bowel preparation.

A meta-analysis showed that ADR did not decrease between high and intermediate quality bowel preparation; however, it was significantly reduced with low-quality bowel preparation [37]. PSSAs are more difficult to detect during a colonoscopy, as they are sessile, mostly proximal in location, and have subtle endoscopic features [15, 16, 37]. Hence, it can be reasonably hypothesized that better quality of bowel preparation should yield higher PSSADR.

Interestingly, our study shows that PSSADR, in fact, did not change significantly with excellent, good, or adequate bowel preparation. SSAs have not just one but several characteristic endoscopic features that aid in their detection. On high resolution while “light endoscopy,” “mucous cap,” “indistinct borders,” and a “cloud-like surface” are the features, which have been validated to assist the endoscopists in the detection of SSAs [38, 39] it can be postulated that some of these features are probably more prominent when the quality of bowel preparation is excellent allowing detection of PSSAs. On the contrary, less than optimal bowel preparation may leave a rim of stool around these flat lesions or the mucous cap accompanying the lesions may appear thicker and endoscopically more prominent permitting identification of SSAs. Our results are consistent with two prior studies, in which quality of the bowel preparation had no impact on serrated polyp detection rate [13, 40]. A limitation of these studies was the documentation of PSPDR and not PSSADR specifically.

A recent study by Clark et al. showed results, which are contrasting from our findings. It showed that any quality of bowel preparation less than high quality (excellent/good quality) was associated with a significant decrease in PSSADR. However, it has limited generalizability as the study population consisted of male veterans and colonoscopies were performed by endoscopists with relatively high ADRs than in general clinical practice. That study included both screening and surveillance colonoscopies, in contrast to our study which included only average risk screening colonoscopies. It also did not permit differentiation between good and excellent quality preparations as both these groups were studied together as a high-quality group.

### 4.4. Fellow Participation

It has been shown in previous studies that fellow participation is associated with improved ADR [41] and small adenoma (<5 mm) detection rates [42]. A stepwise increase in ADR was also noted across the years of gastroenterology fellowship training [43, 44]. As compared to adenomas, sessile serrated polyps are particularly challenging to detect due to their subtle features and proximal location [12, 13]. Currently, there are no reported studies, which have assessed the effect of fellow participation on the PSSADR. Our study showed that overall PSSADR was not significantly different with or without fellow participation.

### 4.5. Endoscopist Specialty and PSSADR

There is a definitive gap in the literature about PSSADR when nongastroenterologists perform colonoscopies [19]. Our study shows that gastroenterologists have a significantly higher PSSADR as compared to nongastroenterologists. Development of CRC by serrated pathway is a relatively newer concept and it is likely that surgeons may not be up to date with the current SSA literature, its identifying features, clinical implications of its detection, complete retrieval, and appropriate surveillance measures. Literature review shows conflicting results when comparing the quality of a colonoscopy between a gastroenterologists and surgeons [45–49]. However, none of these studies have systematically compared PSSADR between these two groups of endoscopists. We recommend that educational measures should be implemented to improve the PSSADR in surgeons, which might help in reducing the occurrence of CRC via serrated pathway.

## 5. Limitations

Some limitations of our study are due to its retrospective nature with potential for incomplete data entry and unmeasured bias. It is known that serrated polyp detection rate varies significantly among endoscopists [1]; however, our study included eighty-four endoscopists from different specialties and we did not calculate PSSADR individually for each endoscopist. Also, we did not take colonoscopy withdrawal time into account, as these were not available. Studies have documented higher PSPDR with longer withdrawal time; however, there are no reported studies showing an association between withdrawal time and PSSADR specifically. Split dose bowel preparation was associated with increased sessile serrated polyp detection rates as compared to single-dose preparation [50]. We could not account for these findings in our study, as information about the method and agents used for bowel preparation was not available. Fellow participation and level of fellowship training are shown to be associated with improved ADRs [41, 44]. In terms of serrated lesions, we showed that fellow participation did not make a significant difference in PSSADR; however, current level of fellowship training was not taken into consideration. This association has not been studied before, and future research should be directed to address this important question.

## 6. Conclusion

According to our study results, PSSADR is significantly higher in Caucasians compared to AAs. Hence, it can be reasonably concluded that serrated pathway leading to CRC might play a far greater role in Caucasians than in AAs. Future research should be directed at identifying risk factors associated with this finding. Gastroenterologist outperformed surgeons in terms of PSSADR. Detection of serrated adenomas might be associated with a significant learning curve, and educational measures should be implemented in surgeons to improve their detection rates.

## Figures and Tables

**Table 1:** Patient characteristics.

Factor	Overall
Number of patients	4151
Patient age (years)	60.0 ± 7.7
*Patient gender*	
Female	2207(53.2)
Male	1944(46.8)
*Race*	
Caucasian	3334(80.3)
African-American	631(15.2)
Other	186(4.5)

**Table 2:** Colonoscopy details.

Factor	Overall
Number of endoscopists	84
Number of procedures/endoscopist	49.41 (22.0, 65.0)
*Specialty of the endoscopist*	
Gastroenterology	54(63.5)
General surgery	9(10.6)
Colorectal surgery	21(24.7)
*Fellow participation*	367(8.8)
*Timing of colonoscopy*	
Morning	2920(70.3)
Afternoon	1231(29.7)
*Bowel preparation quality*	
Excellent	652(15.7)
Good	2675(64.4)
Adequate	824 (19.9)

**Table 3:** Overall and gender-specific ADR, PSPDR, PSSADR, and SSADR.

Factor	Overall	Males	Females
ADR	26.4 ± 11.0	32.7 ± 14.7	22.0 ± 12.7
PSPDR	6.1 ± 5.5	7.0 ± 7.8	5.5 ± 6.6
PSSADR	3.3 ± 3.4	3.9 ± 5.4	2.8 ± 4.1
SSADR (proximal + distal)	4.3 ± 3.9	5.2 ± 6.8	3.7 ± 4.7

ADR: adenoma detection rate; PSPDR: proximal serrated polyp detection rate; PSSADR: proximal sessile serrated adenoma detection rate; SSADR: sessile serrated adenoma detection rate.

**Table 4:** Proximal sessile serrated adenoma detection rate (PSSADR) based on patient gender, timing of the procedure, quality of the bowel preparation, and fellow participation.

Factor	PSSADR	*p* value
*Race*		
Caucasians (overall) (*n* = 3334)	3.7 ± 4.1	**<0.001**
African-Americans (overall) (*n* = 631)	0.96 ± 3.5	
Caucasian males	4.2 ± 6.3	**0.003**
African-American males	1.10 ± 5.0	
Caucasian females	3.4 ± 5.1	**<0.001**
African-American females	0.88 ± 3.3	
*Gender*		
Males (*n* = 1944)	3.9 ± 5.4	0.12
Females (*n* = 2207)	2.8 ± 4.1	
*Procedure time*		
Morning (*n* = 2920)	3.2 ± 4.8	0.71
Afternoon (*n* = 1231)	3.0 ± 5.5	
*Quality of bowel preparation*		
Excellent (*n* = 652)	3.2 ± 13.8	0.92
Good (*n* = 2675)	3.7 ± 5.4	
Adequate (*n* = 824)	3.1 ± 10.1	
*Fellow participation*		
Fellow present (*n* = 367)	2.4 ± 13.4	0.49
Fellow absent (*n* = 3784)	3.5 ± 3.8	
*Endoscopist specialty*		
Gastroenterologist (*n* = 64)	3.9 ± 3.5	0.028
Nongastroenterologist (*n* = 30)	2.2 ± 3.0	

**Table 5:** Adenoma detection rate for gastroenterologists and surgeons.

Factor	Gastroenterologist (*n* = 54)	Surgeon (*n* = 30)	*p* value
Overall	28.8 ± 10.5	22.1 ± 10.8	**0.007**
Males	35.2 ± 14.7	28.2 ± 14.2	**0.037**
Females	24.2 ± 12.4	17.8 ± 12.7	**0.027**

**Table 6:** Detection rates for general surgeons and colorectal surgeons.

Factor	General surgeon (*n* = 9)	Colorectal surgeon (*n* = 21)	*p* value
ADR	19.8 ± 8.7	23.1 ± 11.7	0.46
PSPDR	2.1 ± 2.2	5.8 ± 5.5	0.060
PSSADR	1.2 ± 2.1	2.6 ± 3.3	0.23
SSADR	1.2 ± 2.1	3.8 ± 4.0	0.072

ADR: Adenoma detection rate; PSPDR: proximal serrated polyp detection rate; PSSADR: proximal sessile serrated adenoma detection rate; SSADR: sessile serrated adenoma detection rate.

## References

[1] Hetzel J. T., Huang C. S., Coukos J. A. (2010). Variation in the detection of serrated polyps in an average risk colorectal cancer screening cohort. *The American Journal of Gastroenterology*.

[2] Fearon E. R., Vogelstein B. (1990). A genetic model for colorectal tumorigenesis. *Cell*.

[3] Leggett B., Whitehall V. (2010). Role of the serrated pathway in colorectal cancer pathogenesis. *Gastroenterology*.

[4] O'Brien M. J. (2007). Hyperplastic and serrated polyps of the colorectum. *Gastroenterology Clinics of North America*.

[5] Torlakovic E., Snover D. C. (1996). Serrated adenomatous polyposis in humans. *Gastroenterology*.

[6] Huang C. S., O'Brien M. J., Yang S., Farraye F. A. (2004). Hyperplastic polyps, serrated adenomas, and the serrated polyp neoplasia pathway. *The American Journal of Gastroenterology*.

[7] Goldstein N. S. (2006). Serrated pathway and APC (conventional)-type colorectal polyps: molecular-morphologic correlations, genetic pathways, and implications for classification. *American Journal of Clinical Pathology*.

[8] Arain M. A., Sawhney M., Sheikh S. (2009). CIMP status of interval colon cancers: another piece to the puzzle. *The American Journal of Gastroenterology*.

[9] Spring K. J., Zhao Z. Z., Karamatic R. (2006). High prevalence of sessile serrated adenomas with BRAF mutations: a prospective study of patients undergoing colonoscopy. *Gastroenterology*.

[10] Weisenberger D. J., Siegmund K. D., Campan M. (2006). CpG island methylator phenotype underlies sporadic microsatellite instability and is tightly associated with *BRAF* mutation in colorectal cancer. *Nature Genetics*.

[11] Kambara T., Simms L. A., Whitehall V. L. (2004). *BRAF* mutation is associated with DNA methylation in serrated polyps and cancers of the colorectum. *Gut*.

[12] Snover D. C., Jass J. R., Fenoglio-Preiser C., Batts K. P. (2005). Serrated polyps of the large intestine: a morphologic and molecular review of an evolving concept. *American Journal of Clinical Pathology*.

[13] de Wijkerslooth T. R., Stoop E. M., Bossuyt P. M. (2013). Differences in proximal serrated polyp detection among endoscopists are associated with variability in withdrawal time. *Gastrointestinal Endoscopy*.

[14] Rex D. K., Ahnen D. J., Baron J. A. (2012). Serrated lesions of the colorectum: review and recommendations from an expert panel. *The American Journal of Gastroenterology*.

[15] Snover D., Ahnen D. J., Burt R. W., Odze R. D., Bozman F. T., Carneiro F., Hruban R. H. (2010). Serrated polyps of the colon and rectum and serrated (“hyperplastic”) polyposis. *WHO Classification of Tumours Pathology and Genetics Tumours of the digestive system*.

[16] Kahi C. J., Hewett D. G., Norton D. L., Eckert G. J., Rex D. K. (2011). Prevalence and variable detection of proximal colon serrated polyps during screening colonoscopy. *Clinical Gastroenterology and Hepatology*.

[17] Abdeljawad K., Vemulapalli K. C., Kahi C. J., Cummings O. W., Snover D. C., Rex D. K. (2015). Sessile serrated polyp prevalence determined by a colonoscopist with a high lesion detection rate and an experienced pathologist. *Gastrointestinal Endoscopy*.

[18] Gurudu S. R., Heigh R. I., de Petris G. (2010). Sessile serrated adenomas: demographic, endoscopic and pathological characteristics. *World Journal of Gastroenterology*.

[19] Sanaka M. R., Gohel T., Podugu A. (2014). Adenoma and sessile serrated polyp detection rates: variation by patient sex and colonic segment but not specialty of the endoscopist. *Diseases of the Colon & Rectum*.

[20] U.S. Cancer Statistics Working Group (2017). *United States Cancer Statistics: 1999–2014 Incidence and Mortality Web-based Report*.

[21] Kumbhari V., Behary J., Hui J. M. (2013). Prevalence of adenomas and sessile serrated adenomas in Chinese compared with Caucasians. *Journal of Gastroenterology and Hepatology*.

[22] Kahi C. J., Li X., Eckert G. J., Rex D. K. (2012). High colonoscopic prevalence of proximal colon serrated polyps in average-risk men and women. *Gastrointestinal Endoscopy*.

[23] US Census Bureau (2015). https://www.census.gov/quickfacts/fact/table/US/PST045216.

[24] Parikh M. P., Wadhwa V., Jobanputra Y., Naha K., Thota P. N., Sanaka M. R. (2017). Tu1024 high-risk adenoma detection rate: varies by race and fellow participation but not by timing of colonoscopy. *Gastrointestinal Endoscopy*.

[25] Wallace K., Brandt H. M., Bearden J. D. (2016). Race and prevalence of large bowel polyps among the low-income and uninsured in South Carolina. *Digestive Diseases and Sciences*.

[26] Pietrak S. J., Kang M., Patel D., Colangelo T., Ahmad A. S. (2017). Su1668 risk factors for the development of sessile serrated adenomas in a predominantly African-American, female, obese inner city population. *Gastrointestinal Endoscopy*.

[27] Carethers J. M., Murali B., Yang B. (2014). Influence of race on microsatellite instability and CD8^+^ T cell infiltration in colon cancer. *PLoS One*.

[28] Corley D. A., Jensen C. D., Marks A. R. (2013). Variation of adenoma prevalence by age, sex, race, and colon location in a large population: implications for screening and quality programs. *Clinical Gastroenterology and Hepatology*.

[29] Cordice J. W., Johnson H. (1991). Anatomic distribution of colonic cancers in middle-class black Americans. *Journal of the National Medical Association*.

[30] Chu K. C., Tarone R. E., Chow W. H., Alexander G. A. (1995). Colorectal cancer trends by race and anatomic subsites, 1975–1991. *Archives of Family Medicine*.

[31] Lieberman D. A., Williams J. L., Holub J. L. (2014). Race, ethnicity, and sex affect risk for polyps greater than 9 mm in average-risk individuals. *Gastroenterology*.

[32] Rex D. K., Khan A. M., Shah P., Newton J., Cummings O. W. (2000). Screening colonoscopy in asymptomatic average-risk African Americans. *Gastrointestinal Endoscopy*.

[33] Sanaka M. R., Deepinder F., Thota P. N., Lopez R., Burke C. A. (2009). Adenomas are detected more often in morning than in afternoon colonoscopy. *The American Journal of Gastroenterology*.

[34] Chan M. Y., Cohen H., Spiegel B. M. R. (2009). Fewer polyps detected by colonoscopy as the day progresses at a veteran’s administration teaching hospital. *Clinical Gastroenterology and Hepatology*.

[35] Diamond S., Hoda K. M., Holub J. L., Lieberman D. A., Eisen G. M. (2010). 392 does the time of day predict the yield for significant neoplasia on screening colonoscopy?. *Gastroenterology*.

[36] Parikh M. P., Jobanputra Y., Naha K., Wadhwa V., Thota P. N., Sanaka M. R. (2017). Mo1130 impact of race, timing of colonoscopy and fellow participation on sessile serrated adenoma detection rate (SSADR). *Gastrointestinal Endoscopy*.

[37] Clark B. T., Rustagi T., Laine L. (2014). What level of bowel prep quality requires early repeat colonoscopy: systematic review and meta-analysis of the impact of preparation quality on adenoma detection rate. *The American Journal of Gastroenterology*.

[38] Yark H., López-Cerón M., East J. E. (2013). Endoscopic features of sessile serrated adenomas: validation by international experts using high-resolution white-light endoscopy and narrow-band imaging. *Gastrointestinal Endoscopy*.

[39] Yang H.-J., Lee J. I., Park S.-K. (2017). External validation of the endoscopic features of sessile serrated adenomas in expert and trainee colonoscopists. *Clinical Endoscopy*.

[40] Anderson J. C., Butterly L. F., Robinson C. M., Goodrich M., Weiss J. E. (2014). Impact of fair bowel preparation quality on adenoma and serrated polyp detection: data from the New Hampshire colonoscopy registry by using a standardized preparation-quality rating. *Gastrointestinal Endoscopy*.

[41] Rogart J. N., Siddiqui U. D., Jamidar P. A., Aslanian H. R. (2008). Fellow involvement may increase adenoma detection rates during colonoscopy. *The American Journal of Gastroenterology*.

[42] Buchner A. M., Shahid M. W., Heckman M. G. (2011). Trainee participation is associated with increased small adenoma detection. *Gastrointestinal Endoscopy*.

[43] Qayed E., Shea L., Goebel S., Bostick R. M. (2017). Association of trainee participation with adenoma and polyp detection rates. *World Journal of Gastrointestinal Endoscopy*.

[44] Peters S. L., Hasan A. G., Jacobson N. B., Austin G. L. (2010). Level of fellowship training increases adenoma detection rates. *Clinical Gastroenterology and Hepatology*.

[45] Ko C. W., Dominitz J. A., Green P., Kreuter W., Baldwin L. M. (2010). Specialty differences in polyp detection, removal, and biopsy during colonoscopy. *The American Journal of Medicine*.

[46] Wexner S. D., Forde K. A., Sellers G. (1998). How well can surgeons perform colonoscopy?. *Surgical Endoscopy*.

[47] Bhangu A., Bowley D. M., Horner R., Baranowski E., Raman S., Karandikar S. (2012). Volume and accreditation, but not specialty, affect quality standards in colonoscopy. *The British Journal of Surgery*.

[48] Charbel J. M., Bastawrous A. L., Froese D. (2015). Colon and rectal surgeons: raising the endoscopy bar. *Journal of the American College of Surgeons*.

[49] Pace D., Borgaonkar M., Evans B. (2017). Annual colonoscopy volume and maintenance of competency for surgeons. *Surgical Endoscopy*.

[50] Horton N., Garber A., Hasson H., Lopez R., Burke C. A. (2016). Impact of single- vs. split-dose low-volume bowel preparations on bowel movement kinetics, patient inconvenience, and polyp detection: a prospective trial. *The American Journal of Gastroenterology*.

